# Angry Drivers Take Risky Decisions: Evidence from Neurophysiological Assessment

**DOI:** 10.3390/ijerph16101701

**Published:** 2019-05-15

**Authors:** Shuling Li, Tingru Zhang, Ben D. Sawyer, Wei Zhang, Peter A. Hancock

**Affiliations:** 1 State Key Laboratory of Automotive Safety and Energy, Department of Industrial Engineering, Tsinghua University, Beijing 100084, China; sl-li12@mails.tsinghua.edu.cn; 2 Institute of Human Factors and Ergonomics, College of Mechatronics and Control Engineering, Shenzhen University, Shenzhen 518060, China; zhangtr@szu.edu.cn; 3 Department of Industrial Engineering & Management Systems, University of Central Florida, Orlando, FL 32816, USA; sawyer@ucf.edu; 4 Department of Psychology, University of Central Florida, Orlando, FL 32816, USA; Peter.Hancock@ucf.edu

**Keywords:** anger, driver, event-related potentials, risk sensitivity, decision-making

## Abstract

The present study investigated the risk-taking behaviors of angry drivers, which were coincidentally measured via behavioral and electroencephalographic (EEG) recordings. We manipulated a driving scenario that concerned a Go/No-Go decision at an intersection when the controlling traffic light was in its yellow phase. This protocol was based upon the underlying format of the Iowa gambling task. Variation in the anger level was induced through task frustration. The data of twenty-four drivers were analyzed via behavioral and neural recordings, and P300 was specifically extracted from EEG traces. In addition, the behavioral performance was indexed by the percentage of high-risk choices minus the number of the low-risk choices taken, which identified the risk-taking propensity. Results confirmed a significant main effect of anger on the decisions taken. The risk-taking propensity decreased across the sequence of trial blocks in baseline assessments. However, with anger, the risk-taking propensity increased across the trial regimen. Drivers in anger state also showed a higher mean amplitude of P300 than that in baseline state. Additionally, high-risk choices evoked larger P300 amplitude than low-risk choices during the anger state. Moreover, the P300 amplitude of high-risk choices was significantly larger in the anger state than the baseline state. The negative feedback induced larger P300 amplitude than that recorded in positive feedback trials. The results corroborated that the drivers exhibited higher risk-taking propensity when angry although they were sensitive to the inherent risk-reward evaluations within the scenario. To reduce this type of risk-taking, we proposed some effective/affective intervention methods.

## 1. Introduction

Angry driving is an important concern in our daily lives [1]. In addition, angry driving has become a worldwide concern and a strong focus on research. A report released by the American Automobile Association has corroborated that approximately 80% of drivers express significant levels of anger and/or road rage as part of their normal driving habits [2]. In European countries like the UK, Finland, the Netherlands [3], and Spain [4], and also in some Asian countries like China [5] and Japan [6], all drivers have reported certain levels of anger while driving. Angry can be defined as a strong feeling of annoyance, displeasure, or hostility, and it is an emotional state whose motivation is to warn, intimidate, or attack those who are considered as challenging or threatening [7]. Anger is generally elicited by environments, events, and people that are unpleasant, aversive, or undesirable. Moreover, anger is anticipated to exert a negative effect on one’s judgment and decision-making [8]. Today’s driving environments are complicated and growing more so. Driving worlds include interactions not only among drivers themselves but also with pedestrians and on-road driverless vehicles [9]. Therefore, drivers embedded in these complex circumstances experience many triggers that lead to angry driving. Furthermore, traffic conflicts, confrontations with other vehicles, slowed or stopped traffic, as well as any particular driver’s pre-existing propensity for frustration can all contribute to anger episodes [10,11].

Angry driving has been reported to have a series of deleterious consequences. The closest relationship identified is probably how risky or unsafe driving behaviors are related to anger behind the wheel. For instance, Zhang et al. highlighted that aberrant driving behaviors fully mediate the effects of driving anger on road crash risks [12]. In a study carried out among a sample of Norwegian drivers, Iversen demonstrated that those who scored high in driving anger were more often involved in speeding and ignoring traffic rules [13]. In addition, Dahlen corroborated that driving anger, along with other factors such as sensation seeking, impulsiveness, and the big five factors, is a predictor of unsafe driving behaviors such as driving without using a seatbelt and the losses of vehicle control [14]. Considerable evidence that driving anger contributes to high road accident risks has emerged. Furthermore, Deffenbacher affirmed that drivers with high trait anger experience anger more frequently and intensively when driving and are twice as likely to meet accidents compared with those having low trait anger [15]. In addition, there were also some other human factors that highly correlated to angry driving and traffic offending, such as driving stress, social context and mental health [16,17]. For example, previous studies indicated that driving anger mediated the associations between driving stress, risk predisposition, and traffic sanctions [16]. More importantly, the ‘dislike of driving’ and ‘thrill-seeking’ dimensions of drivers’ stress were related to risky driving behaviors such as speeding on inner city roads [17]. Moreover, driving anger also partially mediated the association between driving experience, hourly intensity, and job stress [16].

Researchers have identified the problem and explored why anger has an impact on risky or unsafe driving behaviors. One possible explanation is that anger reduces drivers’ risk perception, which further affects their risky behaviors, such as tailgating, speeding, racing, and violating traffic laws [5,9,10,11,18]. Other explanations imply that anger exerts influence on drivers’ collective perceptual capacities and overall driving competence [19,20]. In addition, recent studies have focused on the analysis of drivers’ decision-making process, especially those that require speeded response [21]. Emotions, especially anger, are heavily involved in such accelerated decisions [15].

While the abovementioned studies have contributed to an understanding of the working mechanisms of driving anger, most outcomes are mainly from subjective measurements (e.g., questionnaire survey) and behavioral performances. The cognitive decision-making process linked to angry driving has not been fully articulated yet. Moreover, the only neurological study on angry driving was by Techer et al., who contended that anger impacts the attentional process and driving performances by provoking an increase in lateral variations while reducing the amplitude of the visual N1 peak [22]. However, this work mainly focused on the attention allocation of angry drivers; whether anger would change drivers’ behaviors by affecting their decisions on risk-taking must be evaluated. Although risky decision-making can be measured using behavioral responses to risk-related stimuli alone, event-related potentials (ERPs) provide the additional direct and temporally precise measurements of the neural processes involved in decision-making [23]. ERPs are scalp-recorded electrophysiological responses generated by the brain and they are associated with particular internal or external cognitive events (e.g., stimuli, responses, decisions, and feedback). Therefore, ERPs have been used to investigate the sensitivity of various risks in decision-making [24,25,26].

A common ERP component that carries important information for reward processing in decision-making is the feedback-related P300. It is a positive waveform whose peak occurs around 300 to 500 ms following the stimulus onset [27,28,29]. The role of P300 lies on the reward evaluation process [27]. Previous studies have claimed that the feedback-related P300 reflects the extent to which information is motivationally significant [30,31]. In accordance with this, the P300 amplitude varies with the motivational significance of feedback information [32,33] and increases when individuals attribute more meaning to feedback [34]. Moreover, the enhancement of the P300 amplitude in high-risk choices reflects the enhanced motivational significance of risky decisions [31].

Research has been carried out to analyze the decision-making process with the indicators of ERPs in risk-involved decision tasks. For instance, the Iowa gambling task (IGT) is a protocol that simulates real-life decision-making in a laboratory environment. It was originally used to test sensitivity to future consequences and the ability to make decisions following upon damage to the prefrontal cortex [35,36]. Since then, the IGT has also been used to discover the neurological bases for risky decision-making and to interpret the interaction between emotion and decision-making [37,38]. For example, Ba et al. validated that IGT performance was a key factor in predicting risky driving behaviors. The results confirmed that high-risk tendency drivers had lower P300 amplitude, indicating that they were relatively insensitive with the reward process in IGT [21]. This task offers an opportunity to investigate the risk-taking attitude of drivers experiencing different states of anger. Further, the cognitive decision-making process of risk-taking in angry driving has rarely been explored. The recent study by Li and coworkers verified that drivers become insensitive with risk and reward in gambling when angry and make associated risky decisions [39]. Collectively, the research implicated a cognitive pattern, i.e., anger leads to risk and reward insensitivity, and hence results in risky behavior. While in their study, participants did not know the risk for each choice and the experiment design was not a driving-related task. Our study aims to explore whether angry drivers become risk sensitive when the risk is evident and reduce risky behaviors in a driving-related scenario.

In light of the above considerations, our study explicitly sought to distinguish how anger affects drivers’ risk-taking in decision-making using a Go/No-Go situation. We hypothesized that the participants in anger would be sensitive to the inherent risk level and become more risk-averse when risks are evident. Moreover, we used performance analysis and EEG recording and most especially ERP assessment to evaluate this proposition and to construct a comprehensive explanation of such effects at the behavioral and neurological levels of analysis.

## 2. Materials and Methods

### 2.1. Participants

Twenty-eight individuals at a university in the southeast region of the United States volunteered to participate in this experiment. All were native English speakers. During the experiment, the participants made self-reports of their anger status on a seven-point Likert scale ranging from 1 “I am not angry at all” to 7 “I am extremely angry” (i.e., please describe how angry are you feeling now) [40]. These reports were made before the experiment began, before and after they received an anger-inducing manipulation, and after the experiment. Four of the participants were excluded from subsequent analysis because they did not report an effective change in anger from the baseline to the manipulated state (i.e., the postinduction self-report anger needed to be higher than four on the Likert scale and greater than the level of anger reported preinduction). Thus, the data of the twenty-four participants (13 males and 11 females, mean age = 20.8 ± 3.6 years) were analyzed. The mean age of male participants was 19.85 years old (SD = 2.36) and female participants was 21.91 years old (SD = 4.66). All of the participants were undergraduates. All of them had a valid driver license and had sustained that status for more than one year. Their average driving experience was 4.63 (SD = 1.98) years, while 79.17% of them had a driving distance of over 5000 km in the previous year, and all of them drove at least once a week. None had any traffic collisions last year. Furthermore, they were all right-handed and had no history of attention deficit hyperactivity disorder or traumatic brain injury. They received a monetary reward (from $1 to $30) based upon their task performances. This study was approved by the relevant Institutional Review Board (SBE-16-11967) concerning the employment of the experimental participants.

### 2.2. Experiment Design

**Figure 1:**
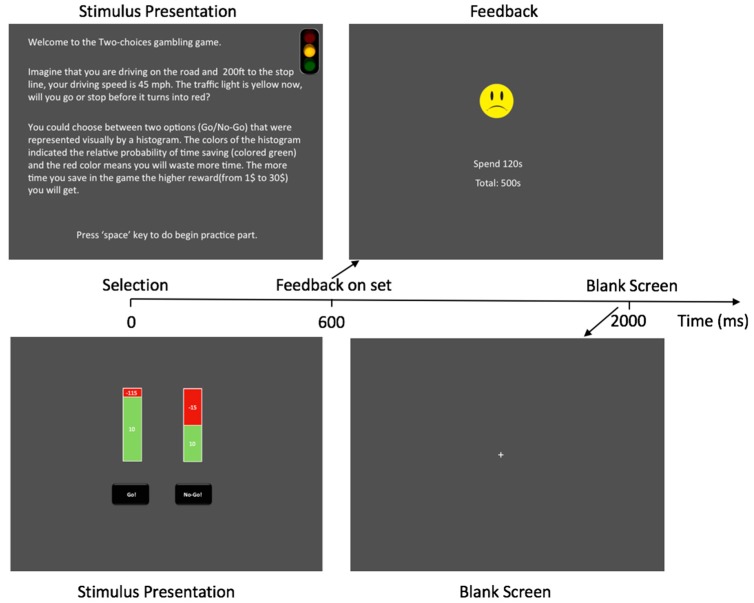
Presentation of the experiment. *Note:* Description of one trial: the participants chose to Go or No-Go, and after 600 ms, the feedback (the positive feedback is time-saving with a smiling face, whereas the negative feedback is time waste along with a sad face) was at the onset and lasted to 2000 ms. Thereafter, a blank screen emerged, and then a new trial began.

The experiment protocol was based on a version of the IGT proposed by Bechara et al. [35]. Some changes were made for this task to conform to our present purpose. First, the experimental scenario was changed to a common driving situation, which featured a Go/No-Go choice at an intersection when the traffic light was yellow. The participants saw a traffic light, and they were told “Imagine that you are driving on the road and are 200 ft (i.e., 60.96 m) from the stop line, and your driving speed is 45 mph (i.e., 72.42 km/h). The traffic light turns yellow. Now, will you go or stop before it turns red?” (See Figure 1.) Here, we chose a distance of 200 ft (i.e., 60.96 m) and a speed of 45 mph (i.e., 72.42 km/h) to simulate driving on an arterial roadway with such active traffic light controls. With the specified distance and speed, the participants had to decide whether to go or stop within 3 s. This is a representative situation in driving, in which drivers must make a quick decision about whether to take the risk or not. Second, in the standard version of the IGT, the participants do not know the exact probability of each card. In our study, the participants knew exactly the chances of winning and losing. One reason is that our study specially examined whether angry individuals would change their risk-taking behaviors when they explicitly knew that an apparent risk was involved.

There were four options (i.e., options A, B, C, and D), and each had a certain probability to save or waste some time. In each trial, the participants were presented with a combination of either options A and B (i.e., AB condition) or options C and D (i.e., CD condition). Table 1 shows the detailed setting of the four options. Taking option A as an example, i.e., choosing to ‘No-Go’ (see Figure 1) at the yellow light, we found that a 50% probability that the light remained yellow and that the participants wasted 15 s for waiting emerged. In addition, we asserted that a 50% probability that the light turned into red and that the participants saved 10 s for successfully avoiding running the red light also emerged. If option B is chosen, which is the ‘Go’ choice in Figure 1, then a 10% probability that the light turned into red and that the participants wasted 115 s for running the red light (representing the punishment for the associated legal infraction) emerged. Furthermore, a 90% probability that the light remained yellow and that the participants saved 10 s also emerged. Options A and B had equal negative expected rewards but differed at the risk level. Option A was a low-risk choice with a smaller standard deviation (SD) (frequent but small losses) compared with option B, and then option B was identified as a high-risk choice with a larger SD (occasional but large losses). In addition, options C and D work in a similar way with A and B, respectively, except that they had positive expected rewards. The design here was to test the participants’ propensity to take high-risk choices while eliminating the effects of expectations.

The amounts of the time saved and wasted for each option were displayed within each histogram. If the participants obtained positive feedback (time saved), then a smiling face would appear on the screen; otherwise, it would be a sad face. The final performance score was time saved minus time wasted, and higher scores were related to higher cash rewards. Moreover, the formal experiment consisted of 200 trials with 100 trials in the first part and 100 trials in the second part. Each part consisted of 50 AB conditions and 50 CD conditions. The condition appeared randomly in the first part, and the sequential of the condition in the second part was the same as the first part.

The stimulus used to induce anger was a gambling task, i.e., anger elicitation gambling task (AEGT). The AEGT was a two-choice task with options M and N in Table 1. The participants chose between the two options for twenty trials in this AEGT, and they were told that they might win or lose in this task. Actually, they kept losing no matter which option they chose.

### 2.3. Experiment Procedure

The participants completed the informed consent and then a demographic questionnaire before the performance element of the study began. We provided practices on the required task prior to recording the actual performance data. Before the first part of the formal experiment, the participants had a baseline anger self-report. Thereafter, they were invited to finish the first part of the IGT test, which included five blocks with twenty trials in each block. After finishing the first part, the participants had a five-minute break during which they were asked to report their anger level (i.e., the preinduction anger level). Subsequently, they were presented with the anger-inducing manipulation (i.e., AEGT). After they finished the AEGT, a postinduction anger self-report was then recorded. If they met the criterion for effective anger induction (see Section 2.1), then they finished the second part of the IGT task. In the end, the participants reported their anger scores after finishing the formal experiment and received a cash reward based on their performances. It took an average of one hour to complete the experiment.

### 2.4. Task EEG Recording

The performance task was programmed in PsychoPy [41] and was presented via a Dell LCD monitor at a resolution of 1024 × 768. The task responses were recorded via the keyboard, mouse, and dedicated response buttons, and the EEG recording was made by using the Advanced Brain Signal equipment (B-Alert X10, Advanced Brain Monitoring, Inc., Carlsbad, CA, USA). In addition, the B-Alert X10 acquired EEG data from the prefrontal, ventral, parietal, and occipital areas with 9 sensor sites (Fz, F3, F4, Cz, C3, C4, POz, P3, and P4). The digitization rate was 256 HZ, and an advanced brain monitoring external sync unit was connected wirelessly to the EEG, thereby providing the timestamp of the packets and response signals. Finally, the amplifier bandpass was 0.1 to 30 Hz.

### 2.5. Data Analysis

In terms of EEG data, time epochs from 200 ms before the feedback onset to 1000 ms after the onset were identified across each trial. The epochs were averaged as every single participant’s ERPs, and then a grand average ERP for the entire sample was calculated.

To quantify P300, we computed the mean amplitude in a time window (400–500 ms) post-onset of feedback for win and loss trials in Fz, Cz, and POz electrodes [42,43]. P300 was analyzed under three variables. The first was anger status (AS), of which we observed a baseline and then a subsequent epoch with increased anger; the second was the feedback valence, which referred to positive (time saved) and negative (time wasted) feedback; and the third was the risk choice (high- and low-risk choices).

For performance, as in previous studies [21,36], we used the percentage of high-risk choices minus low-risk choices, i.e., (B + D) − (A + C) in Table 1. This value represented the risk-taking propensity that was analyzed under two variables. One was the AS, and the other was the performance block (from one to five).

A mixed ANOVA was then conducted to explore the relationships among these identified response variables, and this analysis was conducted using MATLAB (version R2019a, MathWorks, Natick, MA, USA) and SPSS (version 17.0, IBM, Armonk, NY, USA). The significance level was set at 0.05 for all analyses. The partial eta squared ($\eta_{p}^{2}$) was presented as measures of effect size [44].

## 3. Results

### 3.1. Emotion Manipulation

**Figure 2:**
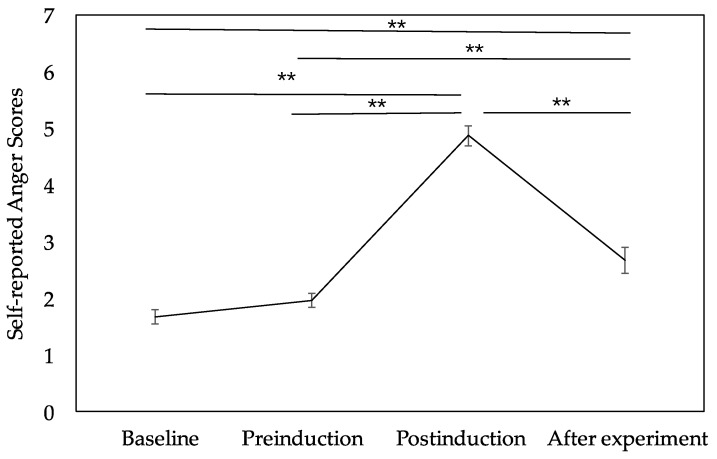
Self-reported anger scores across the four states (baseline, preinduction, postinduction, and after experiment). Error bars indicate the standard error of the mean. * *p* < 0.05, ** *p* < 0.01.

The levels of anger were recorded at four states (i.e., baseline, preinduction, postinduction, and after experiment). Figure 2 shows such data. A significant difference was observed among the four timings for the anger scores, F _(3, 69)_ = 85.21, *p* < 0.001, $\eta_{p}^{2}$ = 0.787. In addition, a paired comparison confirmed that the postinduction anger scores (M = 4.88, SD = 0.90) were significantly higher than those in the preinduction (M = 1.96, SD = 0.62, *p* < 0.001), baseline (M = 1.67, SD = 0.64, *p* < 0.001), and after experiment (M = 2.67, SD = 1.09, *p* < 0.001) states. Moreover, the anger scores in the after experiment state was significantly higher than those in the baseline (*p* = 0.001) and preinduction (*p* = 0.005) states. These results confirmed that the anger manipulation in our experiment was effective and that the affective state persisted throughout the second part of the IGT. Furthermore, no significant difference emerged between the baseline and preinduction states for anger scores. This outcome indicated that the participants had the same effective neutral state before the anger manipulation and that the IGT test did not significantly affect the participants’ anger level.

### 3.2. Performance Data

**Figure 3:**
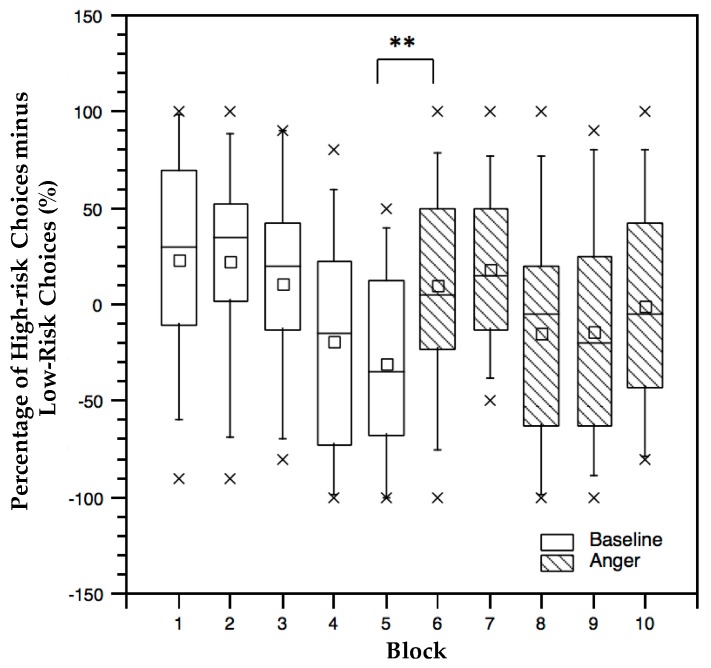
Percentage of high-risk choices minus low-risk choices across blocks. * *p* < 0.05, ** *p* < 0.01.

All of the participants finished ten blocks during the task. The baseline was from block 1 to block 5, and the anger status was from block 6 to block 10. To explore whether anger had an effect on risk-taking behavior, we analyzed the performance data from two aspects. One was the comparison of the two specific blocks just before (block 5) and after (block 6) the participants received anger inducement (Figure 3). This comparison was a way to explore the genesis of risk-taking behavior when anger appeared immediately. In addition, paired *t*-tests were conducted to compare the risk-taking propensity before (block 5) and after (block 6) the anger inducement. Evidently, a significant difference emerged between block 5 (M = −30.42, SD = 10.67) and block 6 (M = 10.00, SD = 10.30, *p* = 0.005). These results claimed that anger actually affected their risk-taking behavior in block 6. A follow-up analysis considered all ten blocks (e.g., baseline and anger). Here, a 2 (AS: baseline vs. anger) * 5 (block: five blocks, with 20 trials in each block) repeated measures ANOVA was used to evaluate the effects of anger and block on the risk-taking propensity. Furthermore, a significant main effect was found for block, F _(4, 96)_ = 9.544, *p* < 0.001, $\eta_{p}^{2}$ = 0.276. No main effect of AS was found. However, a significant two-way interaction emerged between AS and block, F _(4, 96)_ = 5.891, *p* < 0.001, $\eta_{p}^{2}$ = 0.191, see Figure 3. Here, the effects of the block under the baseline and the anger situation were separately analyzed. The results corroborated that the effect of block was significant under the baseline, F _(4, 92)_ = 10.048, *p* < 0.0001, $\eta_{p}^{2}$ = 0.304. Figure 3 exhibits that the trend that the risk-taking propensity decreased across the sequential blocks of the baseline condition (the white bars) emerged. The effect of block under anger was also significant, F _(4, 92)_ = 4.347, *p* = 0.003, $\eta_{p}^{2}$ = 0.159. However, such was not a steadily decreasing trend as was that under the baseline. An increase emerged from block 6 to block 7, and then after a reduction in block 8, the subsequent blocks continued to increase.

### 3.3. ERP Results

**Figure 4:**
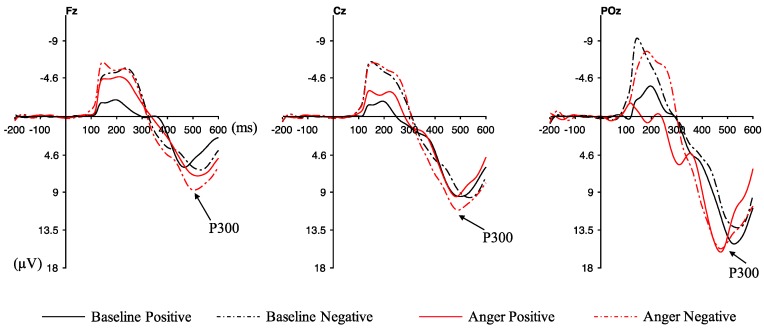
Waveforms of averaged ERPs for the positive and the negative feedback in the electrodes of Fz (**left**), Cz (**middle**), and POz (**right**). *Note:* The feedback stimulus onset occurred at time 0. P300 was calculated from 400 ms to 500 ms after the feedback onset. Negative was plotted up.

Figure 4 shows the waveforms of averaged ERPs in the electrodes of POz, Cz, and Fz. A three-way repeated measures ANOVA was conducted to evaluate the ERP responses, which was a 3 (AS: baseline vs. anger) × 2 (feedback: positive vs. negative) × 3 (electrodes: POz, Cz, and Fz) design. No significant interaction emerged between AS, feedback, and electrodes, and the main effect of AS was significant (F _(1, 23)_ = 5.421, *p* = 0.029, $\eta_{p}^{2}$ = 0.191), with a larger P300 amplitude in the anger state (red lines in Figure 4) than in the baseline. Moreover, the main effect of feedback showed significant (F _(1, 23)_ = 14.371, *p* = 0.001, $\eta_{p}^{2}$ = 0.385) difference for P300 amplitude, and the negative feedback had a larger P300 amplitude than the positive feedback. No significant difference was found for electrodes, while the electrode of POz represented a larger P300 amplitude than Cz and Fz.

**Figure 5:**
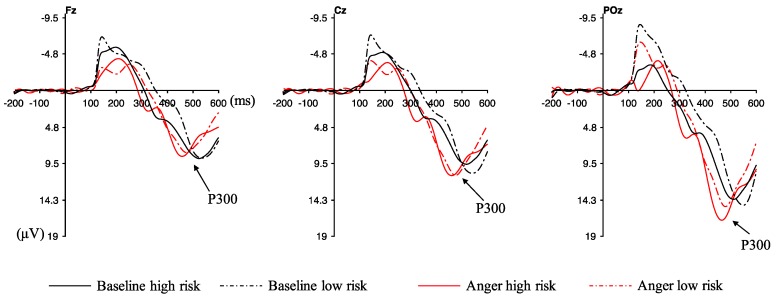
Waveforms of averaged event-related potentials (ERPs) for high- and low-risk choices in the electrodes of Fz (**left**), Cz (**middle**), and POz (**right**). *Note:* The feedback stimulus onset occurred at time 0. P300 was calculated from 400 ms to 500 ms after the feedback onset. Negative was plotted up.

**Figure 6:**
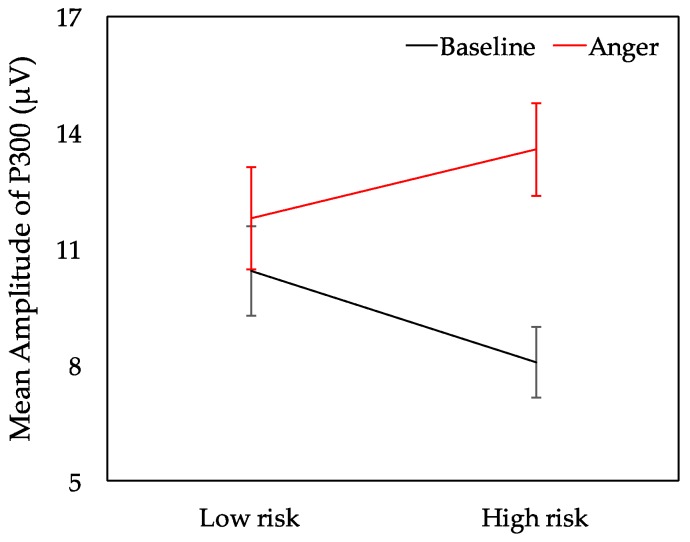
Mean amplitude of P300 for different risk choices under the baseline (**black line**) and anger (**red line**) states. Error bars indicate the standard error of the mean.

Figure 5 exhibits the waveforms of averaged ERPs for different risk choices. A three-way repeated measures ANOVA was conducted to evaluate the ERP responses in the electrodes of POz, Cz, and Fz, which was a 2 (AS: baseline vs. anger) × 2 (risk choice: high risk vs. low risk) × 3 (electrodes: POz, Cz, and Fz) design. A significant interaction emerged between AS and risk choice, F _(1, 23)_ = 4.612, *p* = 0.043, $\eta_{p}^{2}$ = 0.167. Subsequent simple effect analysis corroborated that the significantly greater P300 amplitude was found in high-risk choices than low-risk choices in the anger state (*p* = 0.027) (see Figure 6). By contrast, there was no significant difference between high-risk choices and low-risk choices for the P300 amplitude in the baseline (*p* = 0.07), while there was a trend that low-risk choices evoked larger P300 amplitude. Moreover, the P300 amplitude of high-risk choices was significantly larger in the anger state than the baseline state (*p* = 0.001). To the opposite, the P300 amplitude of low-risk choices showed no significance under anger and baseline states (*p* = 0.953). Furthermore, the main effect of AS was significant (F _(1, 23)_ = 8.080, *p* = 0.009, $\eta_{p}^{2}$ = 0.260), with larger P300 amplitude in the anger state (red lines in Figure 5) than in the baseline. Other main effects or interactions did not reach significance.

## 4. Discussion

We sought to establish whether and how risk taking varies in the different states of drivers’ anger. Through the choice of a daily driving scenario, we were able to evaluate a common driving decision-making process based on our driving-oriented elaboration of the IGT. Previous studies have affirmed that participants showed lower amplitude of P3 and feedback-related negativity when angry, which reflected that they were insensitive to the reward magnitude and unexpected losses. They exhibit a greater risk tendency in anger state than baseline state [39]. Our present research explicitly exposed this risk level to the drivers to establish whether angry drivers would change their risk-taking behaviors by increasing their sensitivity to such risk. Conclusively, the ERP results contended that they were clearly sensitive to high risks when angry. However, drivers did not become commensurately risk-averse. The neurological results and performance outcome mutually support this conclusion.

As the basis of the present study, effective anger induction is essential. The results claimed that anger induction with our anger elicitation gambling task was successful, which was indicated by the significant increase of anger scores from the preinduction to postinduction states. In addition, the preinduction anger score showed no significant difference with the baseline state, which reflected that participants had a similar neutral state during the baseline IGT. The anger scores in the after experiment state was still higher than those in the baseline and preinduction states, which reflected that the affective state persisted throughout the second part of the IGT. The results further validated that the anger emotion can last for quite a while after the triggers disappeared.

In line with previous studies, our findings exhibited that the participants became risk-averse and changed their risk-taking behavior when they were aware of the exhibited risk in the baseline [31]. In this regard, at baseline, risk-taking showed a significant decline trend. We considered this phenomenon to be a reflection of the reward evaluation process and the leaning effect [35,36]. Conversely, the risk-taking increased when anger appeared. In addition, a significant growth emerged from the fifth block to the sixth block, which represented the end of the trials in the baseline and the start of the block under anger, respectively. This result revealed that anger increased the participants’ risk-taking propensity immediately [19,39,45]. Subsequently, after a decline at the eighth block, the risk-taking propensity increased a little late in the trend sequence when angry. The above results were in accordance with some findings in daily driving scenarios. Similarly, Ba et al. verified that many “Go” decisions emerge in dilemma scenarios in daily driving, such as yellow lights [21]. Admittedly, gambling and roadway decisions have similar characteristics: a need for quick and frequent responses, fast feedback, and outcomes that both affect, and are affected by, the emotional state and disposition. Just as angry gamblers show errors in card judgment, angry drivers demonstrate higher speed in driving simulations, shorter times to collisions, higher probability to crash, and self-report more driving violations [14,39,40,46,47].

Neural evidence was derived from the measured P300 component. The main neural findings for P300 were the significant main effects of anger, feedback, and the interaction between anger and risky choice. The participants expressed a higher P300 amplitude when they were angry compared with that in the baseline. Additionally, high-risk choices evoked a larger P300 amplitude than low-risk choices when angry. Moreover, the P300 amplitude of high-risk choices was significantly larger in the anger state than the baseline state. In line with previous studies [48,49], P300 amplitudes were enhanced on negative compared to the positive feedback. A higher P300 amplitude suggests a high motivation, which is in line with previous observations [50]. P300 could indicate the motivational significance of engagement during reward evaluation [28,42,43], and demonstrating that angry drivers engaged more in neural resources than in the baseline is reasonable. The P300 enhancement in high-risk choices also reflected the enhanced motivational significance of risky decisions [31]. The results proved that angry drivers became more motivated with the reward evaluation process and more sensitive to high risks when they were angry [50,51,52]. The neuro findings were different from previous studies. The risk was evident in our study, and the participants became risk-averse in the baseline. They were aware of the risk throughout the experiment and even more sensitive to the risk when angry. However, anger probably affected their judgment on these choices and weakened the learning process, leading to an increasing trend of risk-taking. Moreover, participants might become sensation-seeking when angry, and they preferred to take high risks [53]. They were highly motivated by rewards. In this regard, the participants exhibited high risks to obtain a higher reward when angry. They might pay more attention to timely feedback with high rewards and ignore the long-term optimal decision strategy when angry. There is a possibility that participants might change their preference of the choices or become fearless about the danger when angry. Further studies could add an interview after the experiment to discuss the reasons for their risky behaviors, even if they were aware of the risks. The decision-making process was influenced by multiple cognitive and affective processes [54], whereas the decision-making process, induced by anger, was different in terms of the reward process and risk sensitivity.

Therefore, anger has an impact on drivers’ risk-taking behaviors even when they are sensitive to risks. Both of the results from neurological and behavioral levels support such a conclusion. Behavior could be driven by different valuation systems, and these systems can operate in the domain of rewards and punishments [55]. However, anger could affect this valuation process and lead to risky choices.

## 5. Conclusions

The current work looked to connecting the differential states of driving anger to risk sensitivity directly through reference to the reflections of brain activity. Our study indicated that angry drivers had a supplementary motivation to reward and risk sensitivity compared with that in the baseline. The results corroborated that angry drivers became risk takers although they were sensitive to the risk and reward evaluation process. In this regard, it might be inappropriate to just tell angry drivers ‘It’s dangerous and don’t do that!’ for anger intervention, since drivers took risky actions when they were aware of the risks. Effective intervention methods must be proposed and implemented to reduce such risk-taking propensity. For example, an in-vehicle speech-based agent could be proposed to give feedback when detecting the driver is angry. The agent could tell the drivers something that related to the driving situations and distracts their attention from the anger emotion. Moreover, it could also give some positive feedback to encourage drivers to drive safely. Such techniques could include an anger assessment system whose output is in direct contact with the vehicle controls to provide bounding conditions for drivers in all sub-optional states.

### Limitations of the Study

There were some limitations in this study. First, although self-reporting is widely accepted as a reliable indicator of an individual’s emotional state [56,57], the anger measurement could be improved by adding more common psychological methods like ‘Profile of Mood States’ [58], along with systematic affect detection systems, such as facial expression and neurophysiological data or physiological sensing [59]. Second, due to the requirements of the neurophysiological assessment (i.e., plenty of repetitive tasks and fewer body movements) [25], the present study employed a gambling task instead of a driving task. Previous studies indicated that IGT performance was a key factor in predicting risky driving behaviors. The task we used here still have limitations. For example, the behavior measured in the current study was different from actual driving, which might have concerns on external validity.

Further studies could overcome the limitations of the present study by using multiple psychological methods and simulated driving tasks. Also, factors related to driving anger and traffic violations could be taken into consideration in future studies, such as driving stress, social content, mental workload, and mental health. Moreover, the effectiveness of anger interventions based on the findings in the present study could be tested in a simulated driving environment.

## Figures and Tables

**Table 1:** Probability and value of experiment task choices.

Probability and Value	Go/No-Go Gambling Task				Anger Elicitation Gambling Task	
	No-Go Choices (A)	Go Choices (B)	No-Go Choices (C)	Go Choices (D)	M	N
Time saved	+10	+10	+5	+5	25	30
Time wasted	−15	−115	0	−20	−15	−25
Probability of time saved	50%	90%	50%	90%	0%	0%
Probability of time wasted	50%	10%	50%	10%	100%	100%
Expected value	−2.5	−2.5	2.5	2.5		
Standard deviation	12.5	37.5	2.5	7.5		
Choice type	Low risks with negative expected values	High risks with negative expected values	Low risks with positive expected values	High risks with positive expected values		

*Note:* The expected value was calculated as ‘Probability of time saved × time saved + Probability of time wasted × time wasted.

## References

[1] Galovski T.E., Blanchard E.B. (2004). Road rage: A domain for psychological intervention?. Aggress. Violent Behav..

[2] AAA Foundation (2016). Prevalence of Self-Reported Aggressive Driving Behavior: United States, 2014.

[3] Parker D., Lajunen T., Summala H. (2002). Anger and aggression among drivers in three European countries. Accid. Anal. Prev..

[4] Sullman M.J.M., Gras M.E., Cunill M., Planes M., Font-Mayolas S. (2007). Driving anger in Spain. Personal. Individ. Differ..

[5] Li F., Yao X., Jiang L., Li Y. (2014). Driving anger in China: Psychometric properties of the Driving Anger Scale (DAS) and its relationship with aggressive driving. Personal. Individ. Differ..

[6] McLinton S.S., Dollard M.F. (2010). Work stress and driving anger in Japan. Accid. Anal. Prev..

[7] Kennedy H.G. (1992). Anger and Irritability. Br. J. Psychiatry.

[8] Averill J.R. (1983). Studies on anger and aggression: Implications for theories of emotion. Am. Psychol..

[9] Hancock P.A. (2018). Some Pitfalls in the Promises of Automated and Autonomous Vehicles. Ergonomics.

[10] Zhang T., Chan A.H.S., Ba Y., Zhang W. (2016). Situational driving anger, driving performance and allocation of visual attention. Transp. Res. Part F Traffic Psychol. Behav..

[11] Hancock P.A., Scallen S.F. (1999). The Driving Question. Transp. Hum. Factors.

[12] Zhang T., Chan A.H.S., Xue H., Zhang X., Tao D. (2019). Driving Anger, Aberrant Driving Behaviors, and Road Crash Risk: Testing of a Mediated Model. Int. J. Environ. Res. Public Health.

[13] Iversen H., Rundmo T. (2002). Personality, risky driving and accident involvement among Norwegian drivers. Personal. Individ. Differ..

[14] Dahlen E.R., White R.P. (2006). The Big Five factors, sensation seeking, and driving anger in the prediction of unsafe driving. Personal. Individ. Differ..

[15] Deffenbacher J.L., Deffenbacher D.M., Lynch R.S., Richards T.L. (2003). Anger, aggression, and risky behavior: A comparison of high and low anger drivers. Behav. Res. Ther..

[16] Montoro L., Useche S., Alonso F., Cendales B. (2018). Work Environment, Stress, and Driving Anger: A Structural Equation Model for Predicting Traffic Sanctions of Public Transport Drivers. Int. J. Environ. Res. Public Health.

[17] Öz B., Özkan T., Lajunen T. (2010). Professional and non-professional drivers’ stress reactions and risky driving. Transp. Res. Part F Traffic Psychol. Behav..

[18] Hu T.-Y., Xie X., Li J. (2013). Negative or positive? The effect of emotion and mood on risky driving. Transp. Res. Part F Traffic Psychol. Behav..

[19] Lu J., Xie X., Zhang R. (2013). Focusing on appraisals: How and why anger and fear influence driving risk perception. J. Saf. Res..

[20] Chan M., Singhal A. (2015). Emotion matters: Implications for distracted driving. Saf. Sci..

[21] Ba Y., Zhang W., Peng Q., Salvendy G., Crundall D. (2016). Risk-taking on the road and in the mind: Behavioural and neural patterns of decision making between risky and safe drivers. Ergonomics.

[22] Techer F., Jallais C., Corson Y., Moreau F., Ndiaye D., Piechnick B., Fort A. (2017). Attention and driving performance modulations due to anger state: Contribution of electroencephalographic data. Neurosci. Lett..

[23] Euser A.S., van Meel C.S., Snelleman M., Franken I.H.A. (2011). Acute effects of alcohol on feedback processing and outcome evaluation during risky decision-making: An ERP study. Psychopharmacology.

[24] Bacigalupo F., Luck S.J. (2018). Event-related potential components as measures of aversive conditioning in humans. Psychophysiology.

[25] Luck S.J. (2014). An Introduction to the Event-Related Potential Technique.

[26] Miltner W.H.R., Braun C.H., Coles M.G.H. (1997). Event-related brain potentials following incorrect feedback in a time-estimation task: Evidence for a “Generic” Neural System for Error Detection. J. Cogn. Neurosci..

[27] Goldstein R.Z., Cottone L.A., Jia Z., Maloney T., Volkow N.D., Squires N.K. (2006). The effect of graded monetary reward on cognitive event-related potentials and behavior in young healthy adults. Int. J. Psychophysiol..

[28] Pritchard W.S. (1981). Psychophysiology of P300. Psychol. Bull..

[29] Wu Y., Zhou X. (2009). The P300 and reward valence, magnitude, and expectancy in outcome evaluation. Brain Res..

[30] Nieuwenhuis S., Gilzenrat M.S., Holmes B.D., Cohen J.D. (2005). The Role of the Locus Coeruleus in Mediating the Attentional Blink: A Neurocomputational Theory. J. Exp. Psychol. Gen..

[31] Schuermann B., Endrass T., Kathmann N. (2012). Neural correlates of feedback processing in decision-making under risk. Front. Hum. Neurosci..

[32] Yeung N., Sanfey A.G. (2004). Independent Coding of Reward Magnitude and Valence in the Human Brain. J. Neurosci..

[33] Polezzi D., Sartori G., Rumiati R., Vidotto G., Daum I. (2010). Brain correlates of risky decision-making. NeuroImage.

[34] De Bruijn E.R.A., Mars R.B., Hulstijn W., Ullsperger M., Falkenstein M. (2004). “It wasn’t me… or was it?” How false feedback effects performance. Errors, Conflicts, and the Brain. Current Opinions on Performance Monitoring.

[35] Bechara A., Damasio A.R., Damasio H., Anderson S.W. (1994). Insensitivity to future consequences following damage to human prefrontal cortex. Cognition.

[36] Bechara A., Damasio H., Tranel D., Damasio A.R. (1997). Deciding Advantageously Before Knowing the Advantageous Strategy. Science.

[37] Bechara A., Damasio H., Damasio A.R. (2000). Emotion, Decision Making and the Orbitofrontal Cortex. Cereb. Cortex.

[38] Yang J., Li H., Zhang Y., Qiu J., Zhang Q. (2007). The neural basis of risky decision-making in a blackjack task. Neuroreport.

[39] Li S., Sawyer B., Zhang T., Zhang W., Hancock P. (2018). Anger Leads to Risk Insensitivity in Gambling and Driving.

[40] Newhagen J.E. (1998). TV news images that induce anger, fear, and disgust: Effects on approach-avoidance and memory. J. Broadcast. Electron. Media.

[41] Peirce J.W. (2007). PsychoPy—Psychophysics software in Python. J. Neurosci. Methods.

[42] Gehring W.J., Willoughby A.R. (2002). The Medial Frontal Cortex and the Rapid Processing of Monetary Gains and Losses. Science.

[43] Lu H., Wang Y., Xu S., Wang Y., Zhang R., Li T. (2015). Aggression differentially modulates brain responses to fearful and angry faces: An exploratory study. NeuroReport.

[44] Richardson J.T.E. (2011). Eta squared and partial eta squared as measures of effect size in educational research. Educ. Res. Rev..

[45] Yang Q., Zhao D., Wu Y., Tang P., Gu R., Luo Y. (2018). Differentiating the influence of incidental anger and fear on risk decision-making. Physiol. Behav..

[46] Deffenbacher J.L., Lynch R.S., Filetti L.B., Dahlen E.R., Oetting E.R. (2003). Anger, aggression, risky behavior, and crash-related outcomes in three groups of drivers. Behav. Res. Ther..

[47] Sullman M.J.M. (2006). Anger amongst New Zealand drivers. Transp. Res. Part F Traffic Psychol. Behav..

[48] Frank M.J., Woroch B.S., Curran T. (2005). Error-Related Negativity Predicts Reinforcement Learning and Conflict Biases. Neuron.

[49] Schuermann B., Kathmann N., Stiglmayr C., Renneberg B., Endrass T. (2011). Impaired decision making and feedback evaluation in borderline personality disorder. Psychol. Med..

[50] Balconi M., Crivelli D. (2010). FRN and P300 ERP effect modulation in response to feedback sensitivity: The contribution of punishment-reward system (BIS/BAS) and Behaviour Identification of action. Neurosci. Res..

[51] Bellebaum C., Daum I. (2008). Learning-related changes in reward expectancy are reflected in the feedback-related negativity. Eur. J. Neurosci..

[52] Sato A., Yasuda A., Ohira H., Miyawaki K., Nishikawa M., Kumano H., Kuboki T. (2005). Effects of value and reward magnitude on feedback negativity and P300. Neuroreport.

[53] Dahlen E.R., Martin R.C., Ragan K., Kuhlman M.M. (2005). Driving anger, sensation seeking, impulsiveness, and boredom proneness in the prediction of unsafe driving. Accid. Anal. Prev..

[54] Gutnik L.A., Hakimzada A.F., Yoskowitz N.A., Patel V.L. (2006). The role of emotion in decision-making: A cognitive neuroeconomic approach towards understanding sexual risk behavior. J. Biomed. Inform..

[55] Knutson B., Fong G.W., Adams C.M., Varner J.L., Hommer D. (2001). Dissociation of reward anticipation and outcome with event-related fMRI. NeuroRep. Rapid Commun. Neurosci. Res..

[56] Frijda N.H. (1988). The laws of emotion. Am. Psychol..

[57] Lang P.J. (1995). The emotion probe: Studies of motivation and attention. Am. Psychol..

[58] Shacham S. (1983). A Shortened Version of the Profile of Mood States. J. Personal. Assess..

[59] Jeon M., Roberts J., Raman P., Yim J.-B., Walker B.N. (2011). Participatory Design Process for an In-vehicle Affect Detection and Regulation System for Various Drivers. Proceedings of the 13th International ACM SIGACCESS Conference on Computers and Accessibility.

